# Functional osteoclastogenesis: the baseline variability in blood donor precursors is not associated with age and gender

**DOI:** 10.18632/oncotarget.5575

**Published:** 2015-09-10

**Authors:** Eliana Pivetta, Bruna Wassermann, Pietro Bulian, Agostino Steffan, Alfonso Colombatti, Jerry Polesel, Paola Spessotto

**Affiliations:** ^1^ Division of Experimental Oncology 2, Department of Translational Research, CRO-IRCCS, Aviano, Pordenone, Italy; ^2^ Clinical and Experimental Onco-Hematology Unit, CRO-IRCCS, Aviano, Pordenone, Italy; ^3^ Oncologic Pathology Unit, CRO-IRCCS, Aviano, Pordenone, Italy; ^4^ Unit of Epidemiology and Biostatistics, CRO-IRCSS, Aviano, Pordenone, Italy

**Keywords:** osteoclastogenesis, bone lesions, isolation procedure, CD16, monocytes

## Abstract

Mononuclear osteoclast precursors circulate in the monocyte fraction of peripheral blood and form multinuclear cells with all osteoclastic phenotypic characteristics when cultured in the presence of macrophage colony stimulating factor (M-CSF) and receptor activator of nuclear factor kB ligand (RANKL). The method to obtain osteoclast precursors from peripheral blood is simple but the number of recovered osteoclasts is often largely insufficient for functional analyses. The original aim of this study was to develop a rapid and efficient method that could overcome the donor variability and enrich the osteoclast precursors from a small volume of peripheral blood as a basis for future clinical studies to correlate the differentiation potential of circulating osteoclast precursors with bone lesions in cancer patients. We improved the efficiency of osteoclastogenesis by reducing isolation and purification times and overcame the use of flow cytometry and immunomagnetic purification procedures. In our culture system the osteoclast number was increased several-fold and the precursors were able to reach a full differentiation within seven days of culture. Both age as well as gender differences in osteoclastogenesis efficiency were no longer evident by processing limited volume blood samples with this simple and rapid method.

## INTRODUCTION

The skeleton is a dynamic tissue and maintenance of bone mass is controlled throughout life by osteoblasts, which form new bone, and osteoclasts (OCs), which resorb old bone. OCs are large multinucleated cells, formed by fusion of monocyte/macrophage progenitors [1]. Abnormality in the balance between osteoblast and OC activities results in skeletal disorders like osteopetrosis, osteoporosis and inflammatory bone erosion.

Moreover, osteolytic diseases represent a common cause of morbidity in patients affected by many types of cancer: breast, prostate, thyroid, lung and kidney tumors spread preferentially to the bone, causing cancer associated bone lesions and complications [2–7]. Factors produced by tumors growing in the bone microenvironment could be involved in OC recruitment and differentiation at the stage of fusion and multinucleation of pre-OCs. Investigation of intrinsic properties of human OC precursors (pre-OCs) may help us to understand risk factor of osteolytic disorders including bone metastatic growth. In this perspective a higher availability and/or a more effective functional differentiation of circulating pre-OCs could represent useful information for the prevention or more effective treatments of severe osteolytic lesions.

OCs exert their function only in bone tissue; however, pre-OCs are also present in peripheral blood [8–12]. Bone-resorbing OCs can be matured *in vitro* by culture of monocyte/macrophage precursors in the presence of macrophage colony-stimulating factor (M-CSF) and receptor activator of nuclear factor kappa B ligand (RANKL) [13]. These cytokines are essential and sufficient for differentiation and activation of OCs in normal healthy people [14]. The differentiation of OCs is a complex multistep process that requires commitment to differentiate into pre-OCs, fusion and finally activation to become bone-resorbing terminal cells [15]. OCs specifically express proteins that typify the OC lineage, such as tartrate-resistant acid phosphatase (TRAcP), cathepsin K, and the vitronectin integrin receptor αvβ3 [16, 17].

We focused our interest in developing an efficient culture system for human osteoclastogenesis providing a simple method for the enrichment of pre-OCs. More than one hundred blood samples were assessed and the number of TRAcP+ multinucleated OCs and other OC markers in the presence of M-CSF and RANKL as inducers were evaluated.

## RESULTS

### Variable osteoclastogenesis from blood donors

**Figure 1:**
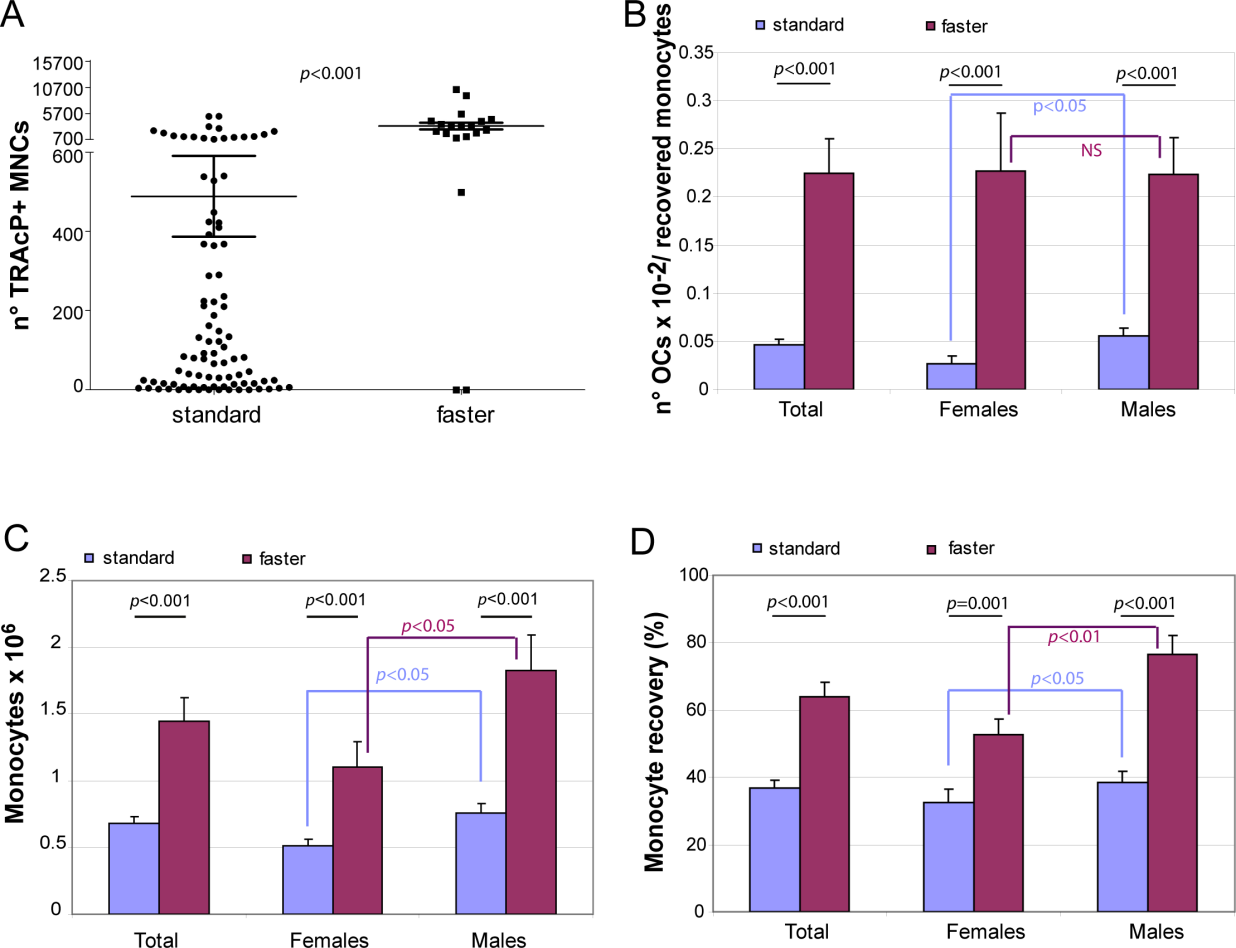
Improvement of osteoclast recovery **A.** TRAcP + MNC formation was valuated for blood samples processed with either standard (*n* = 90) or faster (*n* = 19) method for lympho-mononuclear cellular fraction. The graphs show **B.** OC number normalized for the total number of recovered monocytes, **C.** the total number of the recovered monocytes from 5.5 ml blood samples and **D.** the percentage of the monocyte recovery in the two groups of healthy donors for females and males.

While in age-related disorders the increasing number and the bone resorbing activity of OCs may play an important role in the pathogenesis of bone loss, the effect of aging per se and of gender on OC number is still to be conclusively determined. Despite the efforts to standardize all the procedures adopted, OC formation between different donors (*n* = 90) displayed a great variability. In some cases, at 14 day of culture OCs appeared as really large MultiNuclear Cells (MNCs) with an intense TRAcP staining, in others they were smaller even if multinucleated and TRAcP positive. More importantly, the number of OCs ranged from 0 to more than 5000 (Table 1 and Figure 1). From the analysis of the data reported in Table 1, TRAcP+ MNCs formation resulted gender- (*p* < 0.05, females vs males) but not age-dependent (*p* = NS). Monocytes purified from males differentiated in TRAcP+ MNCs more (3.7 fold) than monocytes obtained from females (632 ± 142 vs 170 ± 58). Younger females produced slightly more TRAcP+ MNCs from peripheral blood than post-menopausal females but this difference was not significant. By converse, monocytes from older males displayed a higher differentiation rate than younger males (915 ± 390 vs 571 ± 151; *p* = NS). Moreover, the analyses of other hematological parameters such as red blood cells count, haemoglobin, platelets count, eosinophil and basophil counts, and the blood group of the donors did not establish any significant correlation with the number of OCs (data not shown).

**Table 1:** Osteoclastogenesis from PBMCs of female and male healthy donors

Sex		n°	Mean age	% MOs in Lympho-MO population	total MOs recovered x10^6(a)^	% MO recovery	total OC/total MOs recovered ×10^−4(a)^	Mean total OC n°
Females	total	28	38±2 (19-63)	29.3±1.7 (16-50)	0.51±0.05 (0.1-1.08)	32.5±3.7 (8.6-100)	2.6±0.66 (0-13.7)	170±58 (0-1216)
	<50	22	33±2 (19-48)	30±2 (16-50)	0.52±0.06 (0.1-1.08)	34.9±4.4 (8.6-100)	2.33±0.62 (0-10.6)	175±71 (0-1216)
	>50	6	56±2 (51-63)	26.7±3.5 (18-40)	0.23±0.06 (0.07-0.4)	23.9±5.2 (10.7-39)	3.6±2.2 (0.13-13.7)	154±78 (2-410)
Males	total	62	41±1 (19-63)	33.7±1.2 (13-52)	0.76±0.07 (0.05-3.7)	38.5±3.1 (4.1-100)	2.76±0.42 (0-12)	632±142 (0-5184)
	<50	51	37±1 (19-49)	33.7±1.3 (13-52)	0.73±0.04 (0.05-3.7)	37±3.3 (4.3-100)	2.64±0.43 (0-12)	571±151 (0-5184)
	>50	11	56±1 (50-63)	33.5±2.9 (18-44)	0.87±0.2 (0.18-3.6)	45.6±8.9 (12.3-100)	6.7±2.56 (0-20.9)	915±390 (0-3210)

(a) from 5.5 ml of peripheral blood

MO, monocyte.

Data are mean ± SE (range values). No significant differences were reported for age-related groups. *p* values are significant for the groups “total MOs recovered” and “Mean total OC number” and are reported as follows:

Total monocytes recovered: *p* < 0.05, total females vs total males

Mean total OC number: *p* < 0.05, total females vs total males

### Faster seeding procedure improves osteoclastogenesis

**Figure 2:**
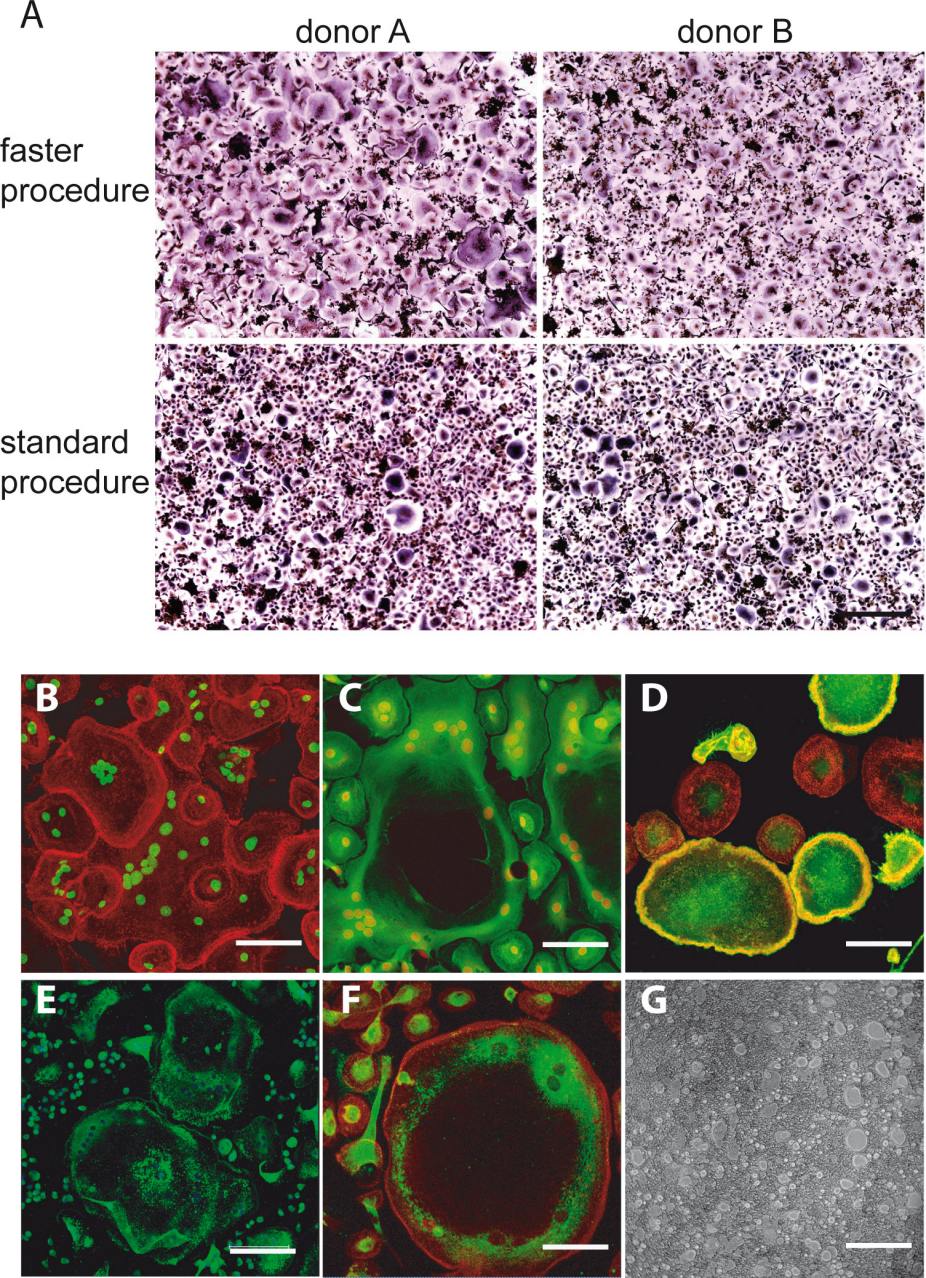
**A.** Direct comparison of the two versions of purification procedure. TRAcP+ MNC formation was visualized after 7-day differentiation period from two different blood donors (donor A and donor B) subjected to both the versions of the purification procedure (upper panel, faster procedure; lower panel, standard procedure). Scale bar, 500 μm. **B.**-**G.** Classical markers of OC differentiation of PBMCs prepared according to “faster” procedure after 7- day of cultures. **B.** Immunofluorescence visualization of the actin rings (actin in red and nuclei in green). **C.** Regular distribution of the tubulin cytoskeleton (tubulin in green and nuclei in red). **D.** Immunofluorescence visualization of αvβ3, mostly associated to the membrane of very large OCs (αvβ3 in green, actin in red). **E.** Immunodetection of cathepsin K (green) and **F.** immunodetection of MMP-9 (MMP-9 in green and actin in red). The positive staining for the two enzymes in areas very close to perinuclear region of the cells is evident, especially for MMP-9. **G.** Representative field of resorption pits of mature OCs on Biocoat surfaces. Bars correspond to 40 μm in **B.**, **C.**, **D.** and **F.** and to 150 μm in **E.** and **G**.

With the “standard” procedure applied above not only the recovery of OCs was variable but also the number of cultures with very few OCs (< 10) was frequent (13 out of 90 samples). We wondered whether the observed low efficiency in OC induction was due to the number of recovered blood OC precursors or was associated to some technical aspects of the purification procedure or to both. To answer this question we skipped May Grunwald-Giemsa staining. This step (around 45 min) had been considered necessary in alternative to FACS analyses and immunomagnetic purification in order to calculate for each donor the precise monocyte percentage in the lympho-monocyte population and to plate an equal number of cells from the different donors. However, after examining the data of the first group of 90 donors reported above, we realized that the percentage of monocytes calculated by May Grunwald-Giemsa staining in the lympho-mononuclear population was usually around 30% (Table 1). Thus, for the subsequent healthy donor group (*n* = 19) we assumed a virtual mean percentage value of 30% and accelerated by one third the time before monocyte seeding. By applying this shortened procedure (“faster” procedure) we surprisingly observed a dramatic improvement in OC formation measured as TRAcP+ MNCs (*p* < 0.001; Figure 1A). First, the mean number of total OCs/monocytes for the faster method versus the standard method was 0.225 ± 0.036 *vs* 0.046 ± 0.006 (*p* < 0.001) (Figure 1B). Importantly, with this procedure not only the age but also the gender difference was no longer detectable (Figure 1B). Second, the absolute number of recovered monocytes obtained after purification was higher both for females and males (Figure 1C, *p* < 0.001). The percentage recovery of monocytes was about twice higher with the latter method (Figure 1D). Thus, the number of OCs obtained was surprisingly much higher than the increased recovery of monocytes, suggesting that the number of monocytes was not the major factor for the increased number of OCs detected. Blood of two different donors was subjected in parallel to the two different procedures maintaining the same concentration of M-CSF and RANKL under both experimental conditions. Figure 2 shows TRAcP+ MNCs after a mere 7-day period of differentiation: for both blood donors, the efficiency in obtaining large and multinucleated OCs was much higher with the modified faster purification procedure.

**Figure 3:**
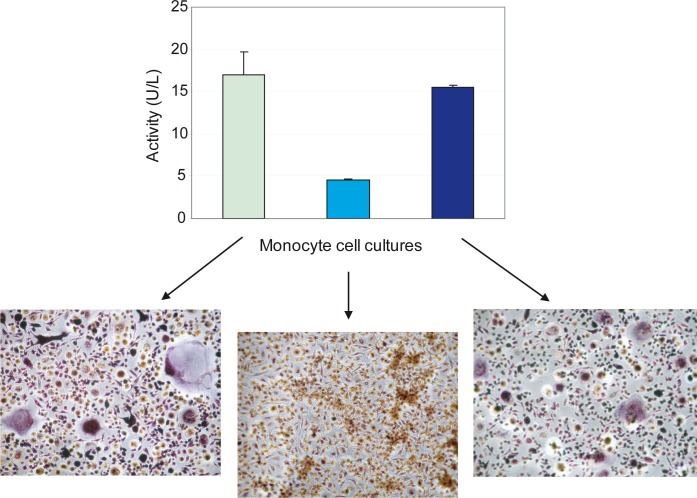
TRAcP activity correlates with osteoclast number TRAcP-5b, performed with BoneTRAP^®^ Assay according to manufacturer's procedure, was reported for three different donors. Monocytes were seeded at 2×10^5^/cm^2^ on a 96-well tissue culture plate and cultured in the presence of M-CSF and RANKL for 7 days. Supernatants were collected, centrifuged and tested. Values are the means ± S.D. The different levels of TRAcP-5b activity correspond to the different intensities in TRAcP staining. Large multinuclear and TRAcP+ cells but also many smaller cells are visible in the representative fields of the images reported below the graph and belonging to donors examined for the TRAcP-5b activity (original magnification, 10x).

Also a number of well-established differentiation biochemical markers typical for mature OCs in addition to TRAcP was examined at 7 days of culture: the actin cytoskeletal organization that represents a characteristic feature of OC functional differentiation [18], the tubulin cytoskeleton, MMP-9 and cathepsin K intracellular localization, αvβ3 integrin distribution and the resorption activity. Representative images in Figure 2 show that the culture conditions are suitable to obtain fully differentiated OCs even at shorter times than usually reported. Finally, we analyzed the supernatants of 7-day-old cultures from three donors. As can be seen in Figure 3, while there was still a variability in the numbers of differentiated OCs even with the faster purification procedure, we could determine a direct correlation between OC density and TRAcP-5b levels.

### Blood cells' parameters

To answer the question whether some blood cells' parameters could contribute to TRAcP+ MNC formation (independently from the procedure used), the donors were divided in two groups on the basis of the median number of OCs obtained. Different cut off values for the two sets of donors were applied (“standard” procedure set, median value = 88; “faster” procedure set, median value = 3088). The donors to whom we applied the “standard” procedure (*n* = 90) were separated into group 1 (G1s) with low efficiency in TRAP+ MNCs formation (total OC number 24 ± 4) (*n* = 45) and group 2 (G2s) with good efficiency (total OC number 954 ± 180) (*n* = 45) (Table 2). The donors to whom the “faster” procedure was applied (*n*= 19) were separated into group 1 (G1f) with lower efficiency (total OC number 1449 ± 338) (*n* = 10) and group 2 (G2f) with higher efficiency (total OC number 5372 ± 865) (*n* = 9) (Table 2). For the OC cultures obtained with the standard procedure the number of monocytes, neutrophils and lymphocytes in peripheral blood was not significantly different in respect of the OCs obtained, whereas there was a highly significant difference (*p* = 2.4×10^−6^) respect to the number of recovered monocytes, suggesting that OC number was proportional to the recovered monocytes and directly dependent on the number of OC precursors isolated (Table 2). For the other set of blood donors (*n* = 19), apart from a highly significant difference in OC number, we did not observe any differences between the two groups for the number of neutrophils, lymphocytes, but for the number of total monocytes (*p* = 0.02). The absolute number of recovered monocytes (*p* = 0.008) was not significant when the percentage of recovered monocytes was taken into account (Table 2). This finding could be explained by the fact that very likely the “faster” purification procedure assured a high monocyte recovery in both groups. The conclusion is that formation of TRAcP+ MNCs likely depends on the good viability and preservation of the small amount of OC precursors circulating in the blood. Still it does not conclusively explain the wide variability in OCs (range 0-10315). One possible explanation is the presence of specific monocyte subpopulations that are differently represented in the donors with higher number of OC precursors.

**Table 2:** Influence on OC induction by peripheral blood populations of “standard” and “faster” version donors

	group (OC range)	n°	total neutrophils (x 10^6^)^(a)^	total lymphocytes (x 10^6^)^a^	total monocytes (x 10^6^)^(a)^	monocytes recovered (x10^6^)^(a)^	% monocyte recovery	OC total n°
STANDARD PROCEDURE	G1 (0-88)	45 (28M-17F)	19.2± 1.1 (9.3-51.7)	12.1± 0.5 (5.5-20.9)	1.8± 0.1 (1.1-3.3)	0.5± 0.04 (0.04-1.1)	25.8±2.1 (4.1-63.4)	24± 4 (0-84)
	G2 (89-6000)	45 (34M-11F)	22.0±1.1 (11.5-41.8)	11.1± 0.3 (7.7-15.9)	2.0± 0.1 (1.1-2.7)	0.9± 0.08 (0.3-2.7)	47.5± 3.8 (23-100)	954± 180 (92-5184)
	p		NS	NS	NS	2.4×10-6	3.0×10-6	1.4×10^™6^
FASTER PROCEDURE	G1 (0-3088)	10 (4M-6F)	20.0± 1.5 (14.3-27)	11.4± 0.6 (8.2-13.7)	1.9± 0.2 (1.1-2.2)	1.2± 0.2 (0.6-2.7)	58.5± 4.0 (36.2-80.6)	1449± 338 (0-3088)
	G2 (3089-10315)	9 (5M-4F)	26.6± 5.6 (12.6-64.3)	13.2± 1.3 (9.3-21.6)	2.6± 0.3 (1.1-4.4)	1.8± 0.3 (0.6-2.9)	69.9± 8.1 (25.8-100)	5372± 865 (3170-10315)
	p		NS	NS	0.02	0.008	NS	5×10^−4^

(a) from 5.5 ml of peripheral blood.

Data are mean value ± SE (range values).

NS, not significant

### Effect of monocyte subpopulations on osteoclast recovery

The use of the “faster” purification method allowed an improved monocyte recovery that was statistically significant compared to the standard procedure (*p* < 0.001) and this resulted in a much higher number of differentiated OCs (Figure 1). Since it was previously reported that TRAcP+ MNCs do not arise from CD16+ monocytes [19], it seemed important to investigate if fewer CD16+ monocytes are present in subjects whose monocytes underwent a more efficient OC differentiation. Thus, to further investigate if the higher number of OC precursors in the mononuclear populations was linked to a specific monocyte subset, we performed a flow cytometry analysis on CD14+ peripheral blood mononuclear cells (PBMCs) [12] immediately after the isolation and washing of the Ficoll-Paque interfaced layers.

**Figure 4:**
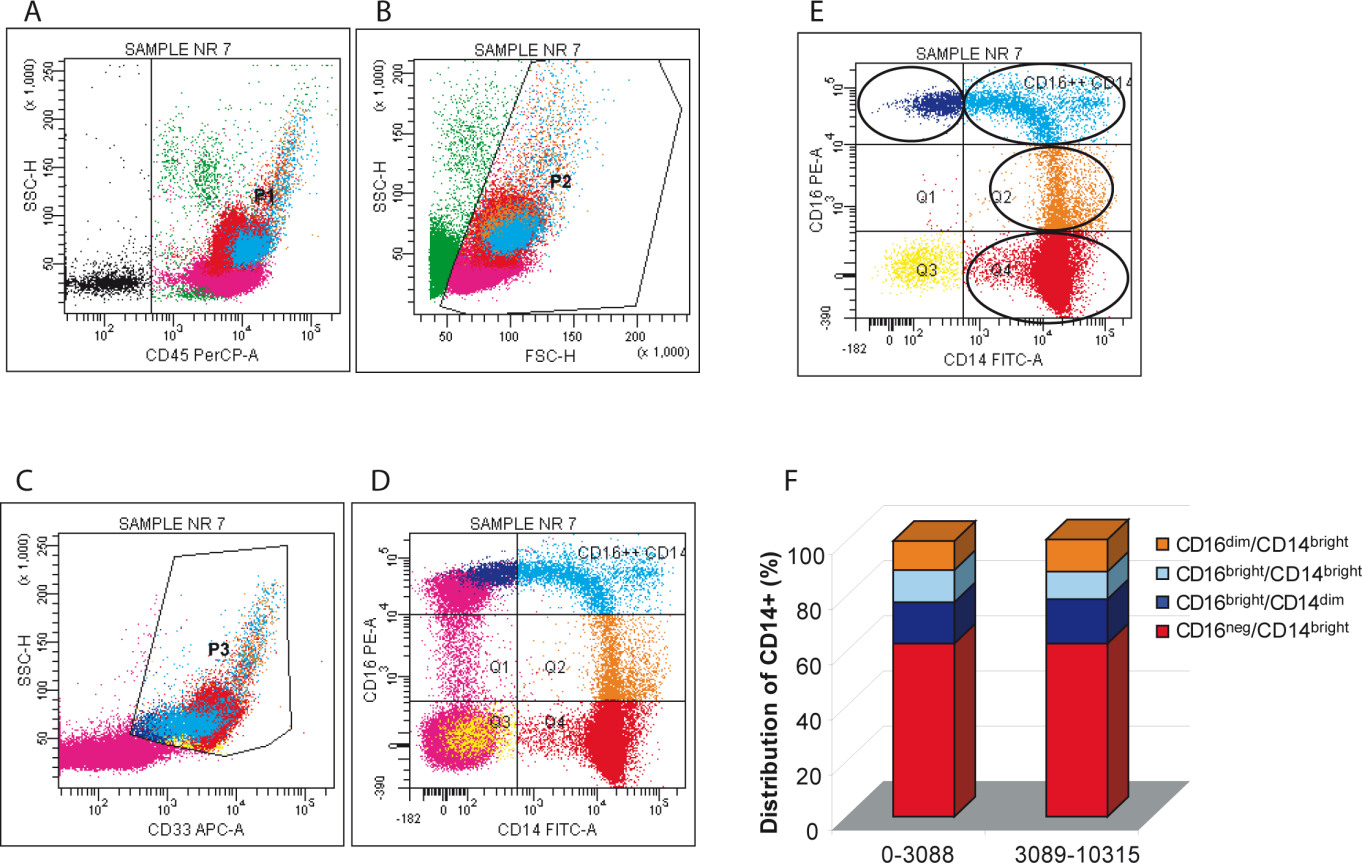
Distribution of monocyte subsets in the lympho-mononuclear fraction: sequential gating strategy **A.** Leukocytes were first identified by CD45 positivity (P1), **B.** dead cells were excluded by scatter signals (P2), **C.** monocytes were defined as CD33 positive cells (both bright and dim) with intermediate side scatter (P3). The P3 region enclosed less than 1% events in unstained controls (not shown). **D.** Quadrants were set on internal control (lymphocytes, purple dots) and an additional region was set on monocyte cells to encompass bright CD16+ expression, defined by the level displayed by NK cells (purple dots). **E.** Monocyte subsets expressing different levels of CD14 and CD16 are shown. The set of 19 donors was subdivided in two groups by the median cut off value. **F.** The distribution of CD14 positive cells on the basis of their CD16 expression level. CD16neg/CD14bright monocytes showed high expression of CD14 and no expression of CD16; CD16dim/CD14bright monocytes also showed a high expression of CD14 and a weaker expression for CD16; CD16bright/CD14bright monocytes showed high expression for both CD14 and CD16; CD16bright/CD14dim monocytes showed a weaker expression of CD14 and a high expression of CD16.

Monocytes were identified by intermediate side scatter and CD33 positivity. Within the CD33 positive monocyte subpopulation we distinguished four sets that differed in CD16 expression levels: CD16^neg^/CD14^bright^, CD16^dim^/CD14^bright^, CD16^bright^/CD14^bright^, and CD16^bright^/CD14^dim^ (Figure 4). By comparing the percentage of different populations of cells in the group with lower number of OCs (range 0-3088) versus the group with more OCs (range 3089-10315) there were no significant differences in any of the monocyte subpopulations analyzed. This finding suggests that since by using the “faster” procedure the number of recovered monocytes was nearly identical between these two groups, i.e. 58.5% and 69.9%, the contribution to a higher efficiency of OC formation depends on intrinsic characteristics of each blood donors. Surprisingly, comparing the percentage of different populations of cells in the female vs male group we obtained significant differences (*p* < 0.05) for CD16^dim^/CD14^bright^ population, being 14.3% in females vs 7.2% in males (data not shown). Again, this finding indicates that in our system the number of CD16 positive cells does not affect the efficiency of osteoclastogenesis.

## DISCUSSION

The aim of the present study was to improve and possibly standardize experimental culture conditions to measure human OC differentiation from small volumes of blood with a rapid and simple method of purification. The necessity to reduce the blood volume is instrumental for the next steps that will follow the present study and is aimed at identifying particular groups of cancer patients at risk of developing more severe osteolytic lesions in their bone metastatic sites. Breast and prostate cancer patients are known to have a great probability to be affected by bone lesions that in most cases are lytic [3, 5, 20].

OC precursors circulate in the monocyte fraction of peripheral blood and form MNCs that express all the phenotypic characteristic of OCs when cultured in the presence of M-CSF and RANKL [13]. The method to obtain monocytes from peripheral blood that will differentiate into OCs is simple but the number of recovered OCs for multi-parametric studies has generally been very low [12, 21]. The low efficiency in OC induction was one of the reasons for the use of cells of different origins and species, i.e. rabbit and mouse OC precursors from bone marrow, to study the biology of OCs [22, 23]. Important knowledge on OC differentiation pathway was obtained also with the use of RAW 264.7, a mouse leukemic monocyte/macrophage cell line that forms mature OCs upon induction [24]. Variable culture conditions also were reported: murine stromal feed layers with human cell progenitors as source of growth and differentiating factors [25], or co-cultures of human OC precursors and osteoblasts in the presence of a number of systemic hormones acting on osteoblastic cells to induce RANKL which in turn led to progenitors differentiation into mature OCs [26, 27].

For our purpose we purified peripheral blood monocytes from a few blood milliliters of a large number *n* = 90) of healthy male as well female donors and evaluated their OC differentiation potential. Monocyte culture density (2.5 × 10^5^ monocytes) represented one of the factors that determined an efficient OC induction (see Figure S1, Supplementary data). Both age and sex were reported to influence the osteoclastogenic potential of PBMCs [25]. In our 90 donors, only gender had a significant impact on OC recovery, whereas age difference in both females and males was irrelevant. Furthermore, the recovery of OC precursors present in the monocyte fraction was substantially increased if the lympho-monocytes population was seeded as soon as possible after washing, i.e. bypassing the interval of time (more or less 45 min) to stain the cells for accurately counting monocyte percentage and thus the number of seeded monocytes. By this simple modification we could improve several-fold the number of OCs (*p* < 0.001) in our cultures and were able to reach a full differentiation at 7 days of culture. Since the number of recovered monocytes is very important for obtaining more OCs, we suggest that a fraction of OC precursors could be lost because monocytes and OC precursors more likely attach to plastic tube even at resting conditions (without stimuli and at 4°C). Thus, the viability and availability of precursors resulted more critical than gender and age of donors; in fact, processing blood samples in parallel with the two versions of the procedure resulted in more TRAcP+ MNCs when lympho-monocytes were seeded immediately after cell isolation. A remarkable aspect of this procedure is represented by the small volume (5.5 ml) of peripheral blood as a basis for future clinical studies: nowadays, the potential of circulating OC precursors with their osteolytic activity is studied using higher amount (20 or 40 ml) obtained with a dedicated taken of a blood sample [28, 29]. In our method, also the leftover of blood samples of patients subjected to routine analyses could be used.

Human peripheral blood monocytes consist of two major subsets, CD16+ and CD16-, that exhibit different chemotaxis activities and potential cytokine production [19, 26, 30, 31]. Komano *et al.* reported that the purified CD16- human peripheral blood monocyte subset, but not the CD16+ subset, differentiates into OCs by stimulation of RANKL in combination with M-CSF [19]. Among the four CD14+ cell populations of healthy donors with variable CD16 expression we could not separate any subgroup that could give rise to more OCs, suggesting that CD16- cells are apparently not more efficient under our experimental conditions. On the other hands, in bone disease patients, CD16 could be a potential marker of OC precursors: in psoriatic arthritis a higher percentage of circulating CD14+ CD16+ cells associated with a higher bone erosion has been reported [32]. It is likely that the percentage of circulating CD14+ CD16+ cells could be determinant in the presence of other than RANKL and M-CFS soluble stimuli: different soluble factors were discovered that can trigger osteoclastogenesis and mainly in inflammatory diseases [33, 34]. Furthermore, other factors might act in concert to program multinucleation process with CD16 [35]. It could be the case of signals delivered by surface ITAM-bearing proteins that regulate the expression of many genes involved in osteoclastogenesis: current data suggest that ITAM-bearing proteins empower cells to acquire a fusion-competent state [36] and recruit Syk kinase [35]. Understanding of the interactions between CD16 and other ITAM-bearing proteins could reveal the contribution of CD16 to osteoclastogenesis at the molecular level.

Differences in OC recruitment were reported in peripheral blood from patients with various bone destructive (osteopenic) diseases, suffering from post-menopausal osteoporosis [28, 37], bone tumors [38] and also periodontitis [39] where PBMCs from patients gave rise to more TRAcP+ MNCs than controls. Spontaneous osteoclastogenesis, for example OC formation without addition of cytokines such as M-CSF and RANKL, was higher in osteoporotic women and arthritis patients [37, 40]. Similarly, spontaneous osteoclastogenesis was increased in cancer patients with bone metastases compared with healthy controls or cancer patients without bone metastases [38].

Another finding of the present study is related to the fact that by improving monocyte recovery both age as well as gender differences were no longer evident, thus suggesting that it is necessary to reconsider some of the data previously reported where these variables affected the number of OCs differentiated *in vitro* [25]. While the absolute number of peripheral blood monocytes remains a significant factor for obtaining the highest number of OCs in both versions of the purification method, with the “faster” version no significant differences in the final total OC number were observed between males and females; on the contrary, in the group of 90 samples processed with the standard procedure OC recovery was significantly higher in males. If differences in the numbers of pre-OCs present in the blood of normal donors do not depend neither on sex nor on age but depend on intrinsic and individual properties of each donor, the consequence is that even more attention on selection and matching of groups of individuals has to be given when comparing osteoclastogenesis in normal vs diseased samples.

In conclusion, investigation of intrinsic properties of human osteoclast precursors may help to understand risk factor of osteolytic disorders. In this perspective a higher availability and/or a more effective functional differentiation of circulating osteoclast precursors could represent useful information for the prevention or more effective treatments of osteolytic lesions.

## MATERIALS AND METHODS

### Reagents for cell cultures

RPMI 1640 medium, glutamine and penicillin/streptomycin were purchased from Cambrex (Milan, Italy), fetal bovine serum from Gibco BRL (Invitrogen, Milan, Italy). Recombinant human sRANKL and recombinant human M-CSF were purchased from PeproTech (London, UK). All chemicals were from Sigma-Aldrich (Milan, Italy) if not otherwise described.

### Cell isolation and cultures

Human peripheral blood was obtained from healthy adult volunteers at the Blood Bank of CRO-IRCCS, National Cancer Institute (Aviano, Italy) in heparinized syringes. Signed informed consent forms approved by the Scientific Director of the Institute were kept on record. Briefly, as previously described [41], peripheral blood samples (5.5 ml) were diluted 1:2 in PBS and overlaid on Ficoll-Paque (Amersham Biosciences, GE Healthcare, UK), and centrifuged at 800 *g* for 20 min. PBMCs were collected into tubes containing PBS, centrifuged, and washed again in PBS. Isolated PBMCs were counted. Monocytes percentage was obtained preparing cytospins and staining cells with May Grunwald-Giemsa dye. In order to select the best concentration of seeded monocytes to obtain the highest induction of OC differentiation monocytes were plated in triplicate in a 96-well plate at 0.5, 1, 2, 5 e 10 × 10^5^ monocytes/well. TRAcP positive cells with three or more nuclei were counted as OCs after 14 days of culture and differentiation curves were obtained (Supplemental Figure 1A). The range of monocyte density with the highest OC induction was 2.0-5.0 × 10^5^ monocytes/well, reaching a plateau at around 2.5 × 10^5^ monocytes/well. We decided to employ 2.0 × 10^5^ monocytes/well that correspond to a 0.7 × 10^6^ monocytes/cm^2^ density. In Supplementary Figure 1 also the morphological appearance of the cultures and the content of OCs are shown. In addition to the number of OCs detected by TRAcP staining, Supplementary Figure 1B shows a corresponding representative dose-response relationship also for the secretion of MMP-9 that is used as marker of OC differentiation and activation [41]. PBMCs were resuspended in an appropriate volume of serum free RPMI medium. Cells were then seeded and incubated for 2 hours in 5% CO_2_, 95% humidified air at 37°C. At the end of the incubation period the wells were washed three times with PBS to remove non-adherent cells. Monocyte purity was more than 95%. Cultures were grown in RPMI 1640 supplemented with 10% heated inactivated FCS, penicillin (100 IU/ml), streptomycin (100 μg/ml), human M-CSF (30 ng/ml) and human sRANKL (40 ng/ml). Cultures were maintained at 37°C and were fed every two-three days with fresh medium and differentiating factors.

### Assays for osteoclast differentiation

*TRAcP staining.* To quantify the formation of TRAcP+ MNCs, monocyte cell cultures were stained for TRAP using Leukocyte Acid Phosphatase kit (Sigma Diagnostics, Milan, Italy), following manufacture's instruction. Cells positive for TRAcP and having more than 3 nuclei were considered as TRAcP+ multinucleated OCs. The optical density was read at 620 nm using an automatic plate reader (GENiosPlus, Tecan Italia, Italy). Each experiment was performed in triplicate and the results expressed as means ± S.E.

*TRAcP-5b assay.* Type-5b tartrate-resistant acid phosphatase (TRAcP-5b) is a specific cytochemical and biochemical marker for osteoclasts and osteoclastic activity. Monocyte cell cultures were analysed using the BoneTRAP^®^ assay (IDS Ltd., Boldon, UK), that is a specific method to detect freshly secreted TRAcP-5b by osteoclasts, according to manufacturer's instructions.

*OC markers.* PBMCs were cultured with human M-CSF (30 ng/ml) and human RANKL (40 ng/ml) for 7 days. Cells were analysed for multinuclearity, actin ring formation, tubulin cytoskeleton, MMP-9 and cathepsin K intracellular localization and αvβ3 integrin distribution. Cells grown on plastic were fixed with 4% paraformaldehyde for 10 min, blocked for non specific binding, permeabilized for 2 min with 0.1% Triton X-100 / 2% FCS / 1% BSA (in PBS) and rinsed with PBS. For cytoskeleton structure visualization, cells were incubated with Alexa 546-conjugated phalloidin (Molecular Probes, Invitrogen) or with FITC-conjugated β-tubulin (Sigma-Aldrich). For cathepsin K, MMP-9 or αvβ3 immunodetection cells were incubated with mouse anti-cathepsin K antibody (Calbiochem, Milan, Italy) or mouse anti-MMP-9 (Chemicon International, Temecula, CA) or mouse anti-αvβ3 (Chemicon International) for 1h at room temperature, followed by chicken anti-mouse Alexa 488-conjugated secondary antibody (Molecular Probes) for 30 min at room temperature. Nuclei were counterstained with Sytox Green Nucleic Acid Stain (Molecular Probes) or propidium iodide for 30 min at room temperature in the dark. After washing with PBS, cells were mounted with Mowiol/0.25% DABCO and observed by confocal microscope. Images were acquired with LEICA TCS SP2 confocal system (Leica Microsystems Heidelberg GmbH, Mannheim, Germany), using the Leica Confocal Software (LCS) and a 40x fluorescence objective on a Leica DM IRE2 microscope.

*Resorption assay.* Monocytes were seeded onto Osteoclast Activity Assay Substrate 96 wells (BioCoat GmbH, Heidelberg, Germany) and cultured for 14 days in complete medium plus M-CSF and RANKL. Cells were removed with NaClO and resorption pits were valued.

### Flow cytometric analysis

PBMCs were prepared as previously described in *Cell Isolation and Cultures*. Aliquots of 0.5×10^6^ cells were incubated for 30 min on ice, in the dark, with the following conjugated antibodies: Fluorescein IsoThyoCyanate (FITC)-conjugated anti-CD14; Peridinin-Chlorophyll-Protein-Cyanine-5.5 (PerCP-Cy5.5)-conjugated anti-CD45; PhycoErithrin (PE)-conjugated anti-CD16, and AlloPhycoCyanin (APC)- conjugated anti-CD33 (BD Pharmigen, BD Biosciences, Italy). Analyses were performed using a FACScalibur flow cytometer (BD Biosciences, Italy).

### Statistical analysis

Data are presented as mean ± S.E. Haematological parameters were compared between groups using ANOVA. *p* value of less than 0.05 was regarded as statistically significant.

## SUPPLEMENTARY MATERIAL FIGURE



## References

[1] Novack DV, Teitelbaum SL (2008). The osteoclast: friend or foe?. Annu Rev Pathol.

[2] Akech J, Wixted JJ, Bedard K, van der Deen M, Hussain S, Guise TA, van Wijnen AJ, Stein JL, Languino LR, Altieri DC, Pratap J, Keller E, Stein GS, Lian JB (2010). Runx2 association with progression of prostate cancer in patients: mechanisms mediating bone osteolysis and osteoblastic metastatic lesions. Oncogene.

[3] Akhtari M, Mansuri J, Newman KA, Guise TM, Seth P (2008). Biology of breast cancer bone metastasis. Cancer Biol Ther.

[4] Cox TR, Rumney RM, Schoof EM, Perryman L, Hoye AM, Agrawal A, Bird D, Latif NA, Forrest H, Evans HR, Huggins ID, Lang G, Linding R, Gartland A, Erler JT (2015). The hypoxic cancer secretome induces pre-metastatic bone lesions through lysyl oxidase. Nature.

[5] Fang J, Xu Q (2015). Differences of osteoblastic bone metastases and osteolytic bone metastases in clinical features and molecular characteristics. Clin Transl Oncol.

[6] Liu H, He J, Yang J (2015). Tumor cell p38 MAPK: A trigger of cancer bone osteolysis. Cancer Cell Microenviron.

[7] Schlampp I, Lang H, Forster R, Wolf R, Bostel T, Bruckner T, Debus J, Rief H (2015). Stability of spinal bone metastases and survival analysis in renal cancer after radiotherapy. Tumori.

[8] Kikuta J, Kawamura S, Okiji F, Shirazaki M, Sakai S, Saito H, Ishii M (2013). Sphingosine-1-phosphate-mediated osteoclast precursor monocyte migration is a critical point of control in antibone-resorptive action of active vitamin D. Proc Natl Acad Sci U S A.

[9] Wang J, Stern PH (2011). Sex-specific effects of estrogen and androgen on gene expression in human monocyte-derived osteoclasts. J Cell Biochem.

[10] Kindle L, Rothe L, Kriss M, Osdoby P, Collin-Osdoby P (2006). Human microvascular endothelial cell activation by IL-1 and TNF-alpha stimulates the adhesion and transendothelial migration of circulating human CD14+ monocytes that develop with RANKL into functional osteoclasts. J Bone Miner Res.

[11] Nicholson GC, Malakellis M, Collier FM, Cameron PU, Holloway WR, Gough TJ, Gregorio-King C, Kirkland MA, Myers DE (2000). Induction of osteoclasts from CD14-positive human peripheral blood mononuclear cells by receptor activator of nuclear factor kappaB ligand (RANKL). Clin Sci (Lond).

[12] Massey HM, Flanagan AM (1999). Human osteoclasts derive from CD14-positive monocytes. Br J Haematol.

[13] Quinn JM, Elliott J, Gillespie MT, Martin TJ (1998). A combination of osteoclast differentiation factor and macrophage-colony stimulating factor is sufficient for both human and mouse osteoclast formation *in vitro*. Endocrinology.

[14] Asagiri M, Takayanagi H (2007). The molecular understanding of osteoclast differentiation. Bone.

[15] Boyce BF, Xiu Y, Li J, Xing L, Yao Z (2015). NF-kappaB-Mediated Regulation of Osteoclastogenesis. Endocrinol Metab (Seoul ).

[16] Faust J, Lacey DL, Hunt P, Burgess TL, Scully S, Van G, Eli A, Qian Y, Shalhoub V (1999). Osteoclast markers accumulate on cells developing from human peripheral blood mononuclear precursors. J Cell Biochem.

[17] Guha M, Srinivasan S, Koenigstein A, Zaidi M, Avadhani NG (2015). Enhanced osteoclastogenesis by mitochondrial retrograde signaling through transcriptional activation of the cathepsin K gene. Ann N Y Acad Sci.

[18] Thirukonda GJ, Uehara S, Nakayama T, Yamashita T, Nakamura Y, Mizoguchi T, Takahashi N, Yagami K, Udagawa N, Kobayashi Y (2015). The dynamin inhibitor dynasore inhibits bone resorption by rapidly disrupting actin rings of osteoclasts. J Bone Miner Metab.

[19] Komano Y, Nanki T, Hayashida K, Taniguchi K, Miyasaka N (2006). Identification of a human peripheral blood monocyte subset that differentiates into osteoclasts. Arthritis Res Ther.

[20] Roato I, D'Amelio P, Gorassini E, Grimaldi A, Bonello L, Fiori C, Delsedime L, Tizzani A, De LA, Isaia G, Ferracini R (2008). Osteoclasts are active in bone forming metastases of prostate cancer patients. PLoS One.

[21] Shalhoub V, Elliott G, Chiu L, Manoukian R, Kelley M, Hawkins N, Davy E, Shimamoto G, Beck J, Kaufman SA, Van G, Scully S, Qi M, Grisanti M, Dunstan C, Boyle WJ, Lacey DL (2000). Characterization of osteoclast precursors in human blood. Br J Haematol.

[22] Tsurukai T, Udagawa N, Matsuzaki K, Takahashi N, Suda T (2000). Roles of macrophage-colony stimulating factor and osteoclast differentiation factor in osteoclastogenesis. J Bone Miner Metab.

[23] Holmes SG, Still K, Buttle DJ, Bishop NJ, Grabowski PS (2004). Chemically modified tetracyclines act through multiple mechanisms directly on osteoclast precursors. Bone.

[24] Xiong Q, Zhang L, Xin L, Gao Y, Peng Y, Tang P, Ge W (2015). Proteomic study of different culture medium serum volume fractions on RANKL-dependent RAW264. 7 cells differentiating into osteoclasts. Proteome Sci.

[25] Jevon M, Sabokbar A, Fujikawa Y, Hirayama T, Neale SD, Wass J, Athanasou NA (2002). Gender- and age-related differences in osteoclast formation from circulating precursors. J Endocrinol.

[26] Sims NA, Gooi JH (2008). Bone remodeling: Multiple cellular interactions required for coupling of bone formation and resorption. Semin Cell Dev Biol.

[27] Roodman GD (2006). Regulation of osteoclast differentiation. Ann N Y Acad Sci.

[28] D'Amelio P, Grimaldi A, Cristofaro MA, Ravazzoli M, Molinatti PA, Pescarmona GP, Isaia GC (2010). Alendronate reduces osteoclast precursors in osteoporosis. Osteoporos Int.

[29] Dalbeth N, Pool B, Stewart A, Horne A, House ME, Cornish J, Reid IR (2013). No reduction in circulating preosteoclasts 18 months after treatment with zoledronate: analysis from a randomized placebo controlled trial. Calcif Tissue Int.

[30] Ancuta P, Rao R, Moses A, Mehle A, Shaw SK, Luscinskas FW, Gabuzda D (2003). Fractalkine preferentially mediates arrest and migration of CD16+ monocytes. J Exp Med.

[31] Ancuta P, Liu KY, Misra V, Wacleche VS, Gosselin A, Zhou X, Gabuzda D (2009). Transcriptional profiling reveals developmental relationship and distinct biological functions of CD16+ and CD16- monocyte subsets. BMC Genomics.

[32] Chiu YG, Shao T, Feng C, Mensah KA, Thullen M, Schwarz EM, Ritchlin CT (2010). CD16 (FcRgammaIII) as a potential marker of osteoclast precursors in psoriatic arthritis. Arthritis Res Ther.

[33] Nakamura I, Takahashi N, Jimi E, Udagawa N, Suda T (2012). Regulation of osteoclast function. Mod Rheumatol.

[34] Wei S, Kitaura H, Zhou P, Ross FP, Teitelbaum SL (2005). IL-1 mediates TNF-induced osteoclastogenesis. J Clin Invest.

[35] Humphrey MB, Lanier LL, Nakamura MC (2005). Role of ITAM-containing adapter proteins and their receptors in the immune system and bone. Immunol Rev.

[36] Humphrey MB, Ogasawara K, Yao W, Spusta SC, Daws MR, Lane NE, Lanier LL, Nakamura MC (2004). The signaling adapter protein DAP12 regulates multinucleation during osteoclast development. J Bone Miner Res.

[37] D'Amelio P, Grimaldi A, Pescarmona GP, Tamone C, Roato I, Isaia G (2005). Spontaneous osteoclast formation from peripheral blood mononuclear cells in postmenopausal osteoporosis. FASEB J.

[38] Roato I, Grano M, Brunetti G, Colucci S, Mussa A, Bertetto O, Ferracini R (2005). Mechanisms of spontaneous osteoclastogenesis in cancer with bone involvement. FASEB J.

[39] Brunetti G, Colucci S, Pignataro P, Coricciati M, Mori G, Cirulli N, Zallone A, Grassi FR, Grano M (2005). T cells support osteoclastogenesis in an *in vitro* model derived from human periodontitis patients. J Periodontol.

[40] Ritchlin CT, Haas-Smith SA, Li P, Hicks DG, Schwarz EM (2003). Mechanisms of TNF-alpha- and RANKL-mediated osteoclastogenesis and bone resorption in psoriatic arthritis. J Clin Invest.

[41] Pivetta E, Scapolan M, Wassermann B, Steffan A, Colombatti A, Spessotto P (2011). Blood-derived human osteoclast resorption activity is impaired by Hyaluronan-CD44 engagement via a p38-dependent mechanism. J Cell Physiol.

