# Supplementary material for: Functional osteoclastogenesis: the baseline variability in blood donor precursors is not associated with age and gender
Source: Oncotarget. 2015 Sep 10;6(31):31889–900. doi: 10.18632/oncotarget.5575 (PMC4741648; doi:10.18632/oncotarget.5575)
Supplement: Supplementary file 1 [file oncotarget-06-31889-s001.pdf]

Functional osteoclastogenesis: the baseline variability in blood donor precursors is not associated with age and gender

Supplementary Material

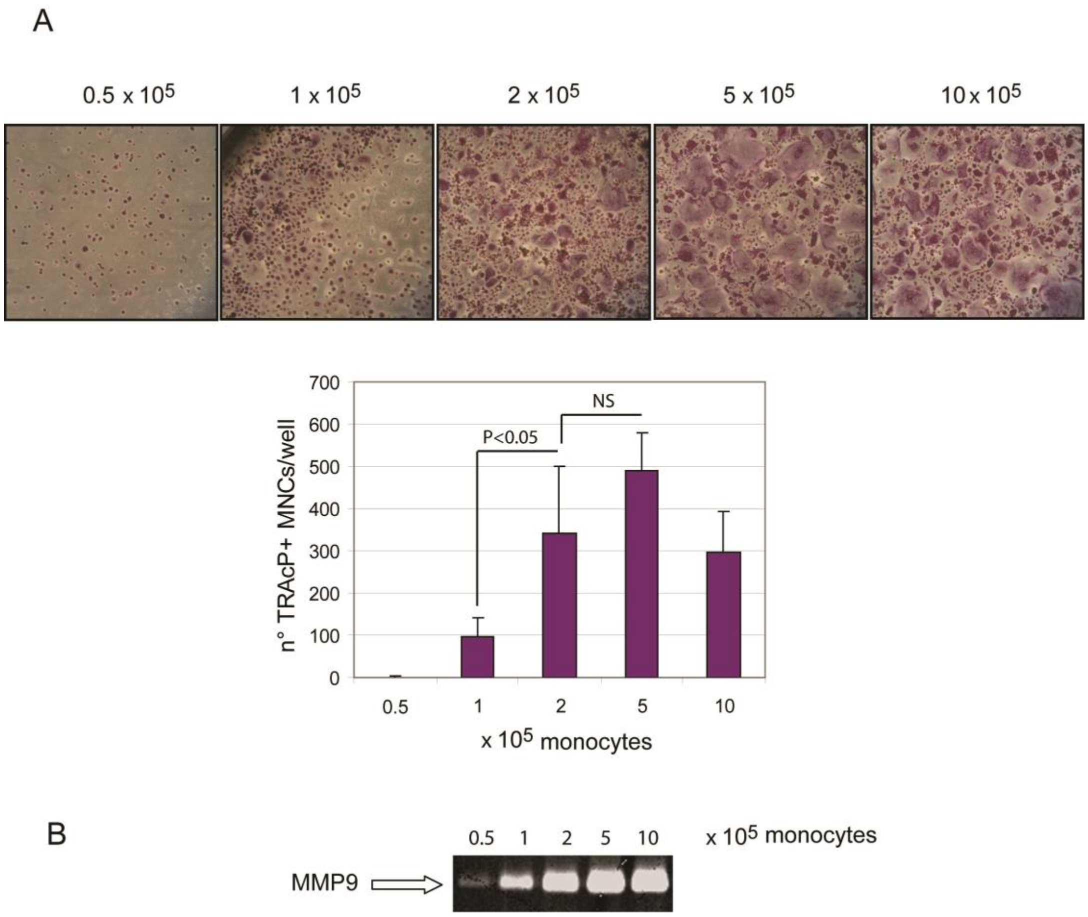

Figure S1. Monocyte density dependent TRAcP+ MultiNuclear Cell formation. (A) Different numbers of monocytes were seeded onto wells of a 96-well plate. After 14 days in the presence of M-CSF and RANKL, the cells were treated for TRAcP staining and TRAcP+ cells with more than three nuclei were counted as OCs. Images of representative fields for the different cell densities are shown (original magnification, 10x). The graph below represents the data (mean OC number ± SD) obtained with three different healthy donors (NS=not significant). The range of monocyte density with the highest OC induction was 2.0-5.0 x 10^5 monocytes/well, reaching a plateau at around 2.5 x 10^5 monocytes/well corresponding to a 0.7x10^6 monocytes/cm^2 density. (B) A representative zymographic analysis showing a corresponding representative dose-response relationship also for the secretion of MMP-9 that is used as marker of OC differentiation and activation. Different numbers of monocytes were allowed to grow in the presence of differentiating agents and after 14 days from the beginning of culture the supernatants were loaded on a 8% SDS-polyacrylamide gel containing 0.1% gelatin. Gels were run under non-reducing conditions and stained in 0.5% Coomassie brilliant blue R-250/30% methanol/10% acetic acid. After destaining, images of Coomassie blue-stained gels were captured.
